# The Prognostic Value of Different Patterns of Neoatherosclerosis and Treatment Modality in Patients With Coronary In‐Stent Restenosis

**DOI:** 10.1155/cdr/2279738

**Published:** 2026-06-17

**Authors:** Youcheng Shen, Ning Gu, Zhijiang Liu, Wei Zhang, Changyin Shen, Xi Wang, Chancui Deng, Qianhang Xia, Yongchao Zhao, Bei Shi

**Affiliations:** ^1^ Department of Cardiology, Affiliated Hospital of Zunyi Medical University, Zunyi, China, zmchospital.com.cn; ^2^ Department of Cardiology, The Third Affiliated Hospital of Zunyi Medical University (The First People′s Hospital of Zunyi), Zunyi, China

**Keywords:** drug-coated balloons, drug-eluting stents, in-stent restenosis, neoatherosclerosis

## Abstract

**Background:**

Neoatherosclerosis (NA) and treatment modality may influence outcomes in patients with in‐stent restenosis (ISR). This study evaluated the prognostic impact of different NA patterns and percutaneous coronary intervention (PCI) strategies guided by optical coherence tomography (OCT).

**Methods:**

We retrospectively analyzed 288 ISR lesions with OCT‐defined NA treated between January 2015 and December 2023. Lesions were classified as lipidic, calcified, or mixed NA. The primary endpoint was 1‐year target lesion failure (TLF), defined as a composite of cardiac death, nonfatal myocardial infarction, and clinically driven target lesion revascularization (CD‐TLR). Cox regression, interaction analysis, and propensity score matching (PSM) were performed.

**Results:**

No significant differences in TLF (*p* = 0.420) or CD‐TLR (*p* = 0.650) were observed among NA patterns. After adjustment for clinical and procedural covariates, DES reimplantation was associated with a lower risk of TLF (hazard ratio [HR] 0.390, 95% confidence interval [CI] 0.157–0.974; *p* = 0.044) and CD‐TLR (HR: 0.341, 95% CI: 0.114–0.925; *p* = 0.039) compared with DCB angioplasty. In patients with lipidic NA, a similar trend was observed (TLF: HR: 0.287; 95% CI: 0.097–0.849; *p* = 0.024; CD‐TLR: HR: 0.171; 95% CI: 0.039–0.750; *p* = 0.019). However, no significant interaction was observed between the NA pattern and treatment modality (*p* for interaction = 0.543).

**Conclusion:**

No significant differences in clinical outcomes were observed across NA patterns. Although DES showed numerically favorable outcomes in lipidic NA, no significant interaction between NA pattern and treatment modality was detected. And these findings are hypothesis‐generating and may relate to the additional mechanical scaffolding and plaque sealing provided by DES in lipidic NA.

## 1. Introduction

Although the iteration of drug‐eluting stent (DES) technologies has significantly improved the effectiveness and safety of percutaneous coronary intervention (PCI) in patients with coronary artery disease, in‐stent restenosis (ISR) is still the main cause of stent failure [1]. For ISR lesions, drug‐coated balloon (DCB) angioplasty or repeat DES implantation has emerged as the most effective therapeutic option and with a Class I indication [2]. Meta‐analysis indicated that in DES‐ISR lesions, repeat DES implantation significantly decreases the rate of target lesion revascularization (TLR) compared with DCB angioplasty [3]. However, the randomized controlled trials (RCTs) [4–6] comparing treatment modalities for ISR focused on angiography‐guided revascularization. Indeed, the isolated coronary angiography supplies little additional information to guide revascularization.

In this respect, optical coherence tomography (OCT) with its superior tissue resolution is widely used to guide revascularization in patients with coronary artery disease, which may provide detailed information with respect to the mechanisms of ISR [7]. Previous investigations have revealed that neoatherosclerosis (NA) is a major cause of ISR, and OCT is recommended to identify NA [8–10]. A few studies illustrated the impact of NA on clinical outcomes in patients with ISR [8, 11]. Chen et al. divided NA into lipidic and calcified NA. Their reports indicated that lipidic NA, but not calcified, was associated with poor clinical outcomes after repeat revascularization [12]. However, previous studies [9, 11, 12] on OCT‐defined NA have primarily focused on isolated neointimal characteristics in relation to clinical outcomes, without examining the association between different morphological patterns of NA and subsequent clinical prognosis under varying treatment modalities. Accordingly, the aim of this study is to investigate whether the OCT‐defined neointimal pattern is correlated to the clinical prognosis of patients undergoing revascularization for ISR and to evaluate whether there is an interaction between different morphological patterns of NA and treatment strategies (DCB or repeat DES) that is related to clinical outcomes.

## 2. Methods

### 2.1. Study Population and Study Endpoint

**Figure 1:**
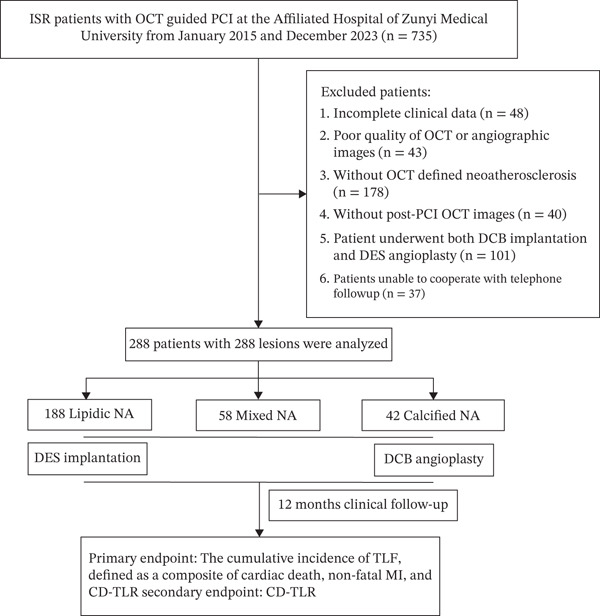
Study flow diagram. CD‐TLR, clinically driven target lesion revascularization; DCB, drug‐coated balloon; DES, drug‐eluting stent; ISR, in‐stent restenosis; NA, neoatherosclerosis; OCT, optical coherence tomography; PCI, percutaneous coronary intervention; TLF, target lesion failure.

This study was a single‐center, retrospective observational study. Between January 2015 and December 2023, 735 patients with ISR undergoing an OCT‐guided PCI at the Affiliated Hospital of Zunyi Medical University were enrolled. The study exclusion criteria were as follows: (1) incomplete clinical data (*n* = 48), (2) poor quality of OCT or angiographic images (*n* = 43), (3) without OCT‐defined NA (*n* = 178), (4) without post‐PCI OCT images (*n* = 40), (5) patient underwent both DCB implantation and DES angioplasty (*n* = 101), and (6) inability to cooperate with telephone follow‐up (*n* = 37). Ultimately, 288 patients with 288 lesions were included in the analysis (Figure 1). This study was approved by the Ethics Committee of the Affiliated Hospital of Zunyi Medical University. When the lesion was severe, pre‐OCT dilation with a diameter less than 2 mm was allowed. Based on the morphological characteristics of pre‐PCI OCT, these patients were divided into lipidic, calcified, and mixed NA groups. The baseline data, including age, sex, coronary risk factors, laboratory data, medicines at discharge, and time from stent implantation to ISR, were collected by experienced physicians from electronic medical record systems. Clinical follow‐up was performed by office visit. For patients who did not attend any outpatient or inpatient follow‐up, telephone contacts would be conducted.

The primary clinical outcome was the cumulative incidence of target lesion failure (TLF) within 1‐year follow‐up, defined as a composite of cardiac death, nonfatal myocardial infarction, and clinically driven target lesion revascularization (CD‐TLR). The secondary endpoint was CD‐TLR. Individual components of the primary endpoint were also assessed separately. Death was considered cardiac unless a noncardiac cause was definitively established [13]. Myocardial infarction was diagnosed when a rise in cardiac biomarkers (preferably troponin) above the 99th percentile upper reference limit was accompanied by at least one of the following: ischemic symptoms, new electrocardiographic changes indicative of ischemia, or imaging evidence of ischemia, which was in accordance with the criteria set forth in the fourth universal definition [14]. CD‐TLR was revascularized for ischemic symptoms linked to the index target lesion.

### 2.2. Angiographic Analysis and PCI Procedures

Patients who had discontinued antiplatelet therapy were loaded with aspirin (300 mg) and a P2Y12 receptor antagonist (clopidogrel 300–600 mg or ticagrelor 180 mg) before the angiography procedure. Coronary angiography was performed following the administration of intracoronary nitroglycerine and prior to PCI. Unfractionated heparin was administered at a weight‐adjusted dose (100 U/kg, with age‐dependent modifications) during the procedure to achieve adequate anticoagulation. The PCI strategies were up to the operator′s discretion but were based on the pre‐PCI OCT findings. Dual antiplatelet therapy—consisting of aspirin plus a P2Y12 inhibitor—was routinely recommended for at least 12 months following PCI [15, 16]. The angiographic features of all patients were analyzed by two veteran cardiologists via the software (Artis VC21C, Siemens AG, Berlin, and Munich). Proximal and distal reference diameters, minimal luminal diameter (MLD) of pre‐PCI and post‐PCI, and percent diameter stenosis (DS) were calculated. Acute gain was defined as the difference between post‐MLD and pre‐MLD.

### 2.3. OCT Imaging and Analysis

**Figure 2:**
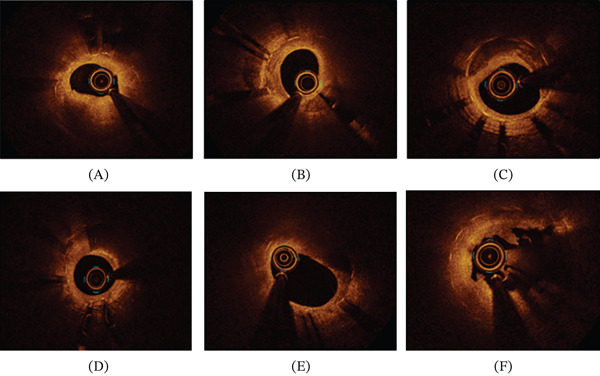
Representative images of optical coherence tomography findings. (A) Lipidic NA. (B) Mixed NA. (C) Calcified NA. (D) Macrophage. (E) TCFA‐like pattern. (F) Intrastent plaque rupture.

OCT imaging was performed using a frequency‐domain OCT system (C7XR, Illumien, or Illumien Optis), following a previously established protocol [17]. Briefly, after coronary angiography, the OCT catheter was advanced across the ISR lesion, and contrast was then injected through the guiding catheter at a flow rate of 3–4 mL/s while simultaneous automated pullback was initiated at a speed of 180 frames/s. All acquired OCT images were analyzed by two trained investigators (Chancui Deng and Zhijiang Liu), who were blinded to angiographic and clinical data. The typical OCT images are shown in Figure 2.

Quantitative neointimal assessment was performed at the minimal lumen area (MLA) site. The proximal and distal reference segments were identified as the sites with the largest lumens within 5 mm proximal and distal to the stented segment. The following parameters were automatically quantified: reference lumen diameter, reference lumen area (proximal and distal), pre‐ and post‐PCI MLA, and area stenosis (AS). Maximal neointimal thickness was the distance between the lumen and the stent strut at the MLA site. Acute luminal area gain was calculated as the difference between the post‐PCI and pre‐PCI MLA. Stent and lumen contours were automatically delineated; manual corrections were applied when suboptimal automated detection occurred.

A qualitative image assessment was performed at the minimum lumen area site as well as at 5 mm proximal and 5 mm distal locations. The neointimal tissue was delineated as the area bounded by the luminal and stent contours. The primary plaque type was first determined at the location of the minimum lumen area. If any lipidic or calcified plaque was observed in at least three consecutive frames, then it was considered NA. Importantly, when both lipidic and calcified components were present and each fulfilled the criterion of ≥ 3 consecutive frames, the lesion was classified as mixed NA, irrespective of their relative proportions. Lesions with purely homogeneous neointima, typically representing fibrous tissue, were not considered as NA and were excluded from the analysis. Findings from these adjacent segments were recorded for descriptive analysis but were not used for primary NA classification. Lipidic plaque was characterized by pronounced signal attenuation with indistinct, diffuse borders [18]. Calcified plaque was defined by regions of low signal intensity and well‐delineated borders. If lipidic and calcified NA both existed in the same stenotic segment, plaque type was considered mixed. The interobserver and intraobserver variabilities for observing ISNA were *k* = 0.878 and *k* = 0.889, respectively; for lipidic plaque, they were *k* = 0.836 and *k* = 0.858, respectively; and for calcified plaque, they were *k* = 0.913 and *k* = 0.933, respectively. Thin cap fibroatheroma (TCFA) was defined as a lipidic plaque with the thinnest part of the fibrous cap < 65 *μ*m and a maximum lipid arc > 180 [19]. Macrophages were identified as well‐demarcated, high‐intensity punctate or confluent regions that exhibited signal intensity exceeding the surrounding background speckle noise upon qualitative visual assessment [17].

### 2.4. Statistical Analysis

Continuous variables are expressed as mean ± standard deviation or median (interquartile range) based on their distribution. Categorical variables are summarized as counts and percentages. Group comparisons were performed using the analysis of variance or the Kruskal–Wallis test for continuous variables and the Pearson chi‐square test or Fisher′s exact test (when expected cell frequencies were below 5) for categorical variables.

The time‐to‐event risk was estimated by the Kaplan–Meier method and was compared using the log‐rank test for each clinical outcome. Hazard ratios (HRs) and corresponding two‐sided 95% confidence intervals (CIs) were derived from Cox proportional hazard regression models. Considering the number of clinical events was relatively limited, this may raise concerns regarding potential model overfitting in multivariable analyses. To address this issue, we adopted a parsimonious modeling strategy by deliberately restricting the number of covariates included in the final Cox regression models. Specifically, only a limited set of clinically relevant variables, including age, diabetes mellitus, and representative cardiovascular risk factors, were selected alongside the primary variables of interest (treatment modality and NA phenotype). This approach was chosen to maintain a more appropriate events‐to‐variable ratio and to enhance the stability and interpretability of the model estimates. Importantly, the main findings remained consistent across sensitivity analyses using alternative model specifications, supporting the robustness of the observed associations despite the limited number of events. Nevertheless, the results of the multivariable analyses should be interpreted with caution, and further studies with larger sample sizes and more events are warranted to validate our findings. And the multivariable model was also used to compare the outcomes between DCB and DES within each OCT‐defined NA subgroup. To test whether the treatment effect differed according to NA patterns, an interaction term between treatment modality (DES vs. DCB) and NA patterns was incorporated into the Cox proportional hazards model. The significance of effect modification was assessed using the *p* value for interaction.

To minimize potential selection bias due to the nonrandomized and operator‐dependent treatment allocation, propensity score matching (PSM) was performed. The propensity score for treatment with DES versus DCB was estimated using a multivariable logistic regression model incorporating clinically relevant baseline variables, including demographic characteristics, cardiovascular risk factors, clinical presentation, angiographic features, and OCT‐derived parameters (Tables S6, S7, and S8). Patients were matched in a 1:1 ratio using nearest neighbor matching without replacement, with a caliper width of 0.2 of the standard deviation of the logit of the propensity score. In the matched cohort, Cox proportional hazards models with robust sandwich variance estimators were used to account for clustering within matched pairs. All tests were set at a two‐sided *p* value of < 0.05. Statistical analysis was performed using SPSS Version 26.0 software (IBM, Armonk, New York, United States) and R software (Version 4.3.3; R Foundation for Statistical Computing, Vienna, Austria).

## 3. Results

### 3.1. Clinical and Procedural Characteristics

A total of 288 patients with NA undergoing PCI were included, with one lesion being imaged/treated per patient. Based on the morphological characteristics of pre‐PCI OCT, patients were categorized into lipidic NA (*n* = 188), calcified NA (*n* = 42), and mixed NA (*n* = 58) groups. Baseline clinical, angiographic, and procedural characteristics according to different patterns of NA are shown in Tables 1 and 2. The time from stent implantation to ISR in calcified NA was longer than those with lipidic and mixed NA. There were no relevant differences in the baseline and procedural characteristics except for the time from stent implantation to ISR among the three groups.

**Table 1:** Clinical characteristics according to the different patterns of neoatherosclerosis.

	Total (*n* = 288)	Lipidic NA (*n* = 188)	Calcified NA (*n* = 42)	Mixed NA (*n* = 58)	*p* value
Age (years), M ± SD	63.0 ± 10.8	62.2 ± 11.1	64.4 ± 10.1	64.9 ± 10.0	0.187
Male, *n* (%)	234 (81.3)	154 (81.9)	31 (73.8)	49 (84.5)	0.372
Hypertension, *n* (%)	176 (61.1)	110 (58.5)	29 (69.0)	37 (63.8)	0.402
Diabetes mellitus, *n* (%)	85 (29.5)	51 (27.1)	14 (33.3)	20 (34.5)	0.473
Dyslipidemia, *n* (%)	90 (31.3)	54 (28.7)	15 (35.7)	21 (36.2)	0.447
CKD, *n* (%)	41 (14.2)	22 (11.7)	6 (14.3)	13 (22.4)	0.125
Stroke, *n* (%)	10 (3.5)	7 (3.7)	1 (2.4)	2 (3.4)	1.000
Smoking, *n* (%)	154 (53.5)	100 (53.2)	23 (54.8)	31 (53.4)	0.983
Previous MI, *n* (%)	38 (13.2)	24 (12.8)	3 (7.1)	11 (19.0)	0.217
Time from stent implantation to ISR, M (IQR) (month)	54.0 (34.3, 72.0)	54.0 (24.0, 60.0)	78.0 (31.0, 108.0)	54.0 (54.0, 60.0)	< 0.001

Laboratory data					

TC (mmol/L), M ± SD	4.19 ± 1.37	4.16 ± 1.49	4.25 ± 1.11	4.26 ± 1.08	0.852
TG (mmol/L), M ± SD	2.13 ± 1.48	2.17 ± 1.55	2.08 ± 1.45	2.05 ± 1.19	0.863
LDL‐C (mmol/L), M ± SD	2.49 ± 0.87	2.49 ± 0.94	2.45 ± 0.70	2.59 ± 0.75	0.742
HDL‐C (mmol/L), M ± SD	1.10 ± 0.25	1.08 ± 0.23	1.10 ± 0.27	1.14 ± 0.28	0.111
FBG (mmol/L), M ± SD	6.04 (5.05, 7.82)	6.10 (4.88, 7.45)	5.65 (5.05, 7.80)	6.31 (5.28, 9.00)	0.196
Uric acid (*μ*mol/L), M ± SD	387.9 ± 101.0	390.4 ± 100.8	387.9 ± 113.2	376.7 ± 80.8	0.783
eGFR (mmol/L), M ± SD	82.5 ± 24.7	84.3 ± 23.5	79.2 ± 29.7	75.4 ± 24.2	0.658
Hemoglobin (g/L), M ± SD	139.6 ± 19.2	140.4 ± 19.1	138.8 ± 16.0	137.5 ± 23.5	0.625
EF (%), M ± SD	52.8 ± 10.4	52.1 ± 10.8	54.3 ± 9.4	53.7 ± 9.6	0.289
NT‐proBNP (pg/mL), M (IQR)	199.0 (89.3, 503.0)	198.5 (85.3, 537.8)	216.0 (66.5, 748.8)	146.0 (85.2, 405.0)	0.611
Hs‐cTnT (ng/mL), M (IQR)	13.9 (8.7, 53.1)	13.1 (8.6, 50.7)	20.1 (10.1, 84.1)	12.6 (8.6, 28.6)	0.217

Clinical presentation on admission					

Stable angina, *n* (%)	66 (22.9)	47 (25.0)	8 (19.0)	11 (19.0)	0.514
UA, *n* (%)	177 (61.5)	113 (60.1)	31 (73.8)	33 (56.9)	0.186
NSTEMI, *n* (%)	24 (8.3)	17 (9.0)	1 (2.4)	6 (10.3)	0.321
STEMI, *n* (%)	21 (7.3)	11 (5.9)	2 (4.8)	8 (13.8)	0.131

Medicines at discharge					

Aspirin, *n* (%)	256 (88.9)	168 (89.4)	49 (84.5)	39 (92.9	0.416
P2Y12 inhibitor, *n* (%)	248 (86.1)	157 (83.5)	39 (92.9)	52 (89.7)	0.195
*β*‐Blocker, *n* (%)	162 (56.3)	107 (56.9)	22 (52.4)	33 (56.9)	0.861
Statin, *n* (%)	260 (90.3)	171 (91.0)	38 (90.5)	51 (87.9)	0.733

Abbreviations: CKD, chronic kidney disease; eGFR, estimated glomerular filtration rate; FBG, fasting blood glucose; HDL‐C, high‐density lipoprotein cholesterol C; Hs‐TnT, hypersensitive cardiac troponin T; LDL‐C, low‐density lipoprotein cholesterol C; M (IQR), median (interquartile range); M ± SD, mean ± standard deviation; NSTEMI, non‐ST‐elevation myocardial infarction; NT‐proBNP, N‐terminal pro‐B‐type natriuretic peptide; STEMI, ST‐elevation myocardial infarction; TC, total cholesterol; TG, triglyceride.

**Table 2:** Angiographic and procedural characteristics according to the different patterns of neoatherosclerosis.

	Total (*n* = 288)	Lipidic NA (*n* = 188)	Calcified NA (*n* = 42)	Mixed NA (*n* = 58)	*p* value
Lesion location					0.929
LAD, *n* (%)	169 (58.7)	108 (57.4)	27 (64.3)	34 (58.6)	
RCA, *n* (%)	88 (30.6)	59 (31.4)	12 (28.6)	17 (29.3)	
LCX, *n* (%)	31 (10.8)	21 (11.2)	3 (7.1)	7 (12.1)	
Predilation, *n* (%)	251 (81.0)	167 (88.8)	37 (88.1)	47 (81.0)	0.295
Scoring balloon, *n* (%)	41 (14.2)	22 (11.7)	8 (19.0)	11 (19.0)	0.241
Cutting balloon, *n* (%)	44 (15.3)	26 (13.8)	7 (16.7)	11 (19.0)	0.614
DES, *n* (%)	120 (41.7)	81 (43.1)	17 (40.8)	22 (37.9)	0.774
DCB, *n* (%)	168 (58.3)	107 (56.9)	25 (59.5)	36 (62.1)	0.774
DCB diameter (mm), M ± SD	3.09 ± 0.44	3.09 ± 0.46	3.17 ± 0.38	2.96 ± 0.39	0.169
DCB length (mm), M ± SD	23.6 ± 5.9	23.5 ± 6.0	22.2 ± 5.6	26.2 ± 5.1	0.029
DCB inflation pressure (atm), M ± SD	11.1 ± 3.3	11.4 ± 3.6	10.2 ± 2.4	11.3 ± 3.2	0.182
DCB inflation time (s), M ± SD	67.3 ± 17.2	66.9 ± 16.5	68.3 ± 17.6	67.8 ± 19.5	0.896
DES diameter (mm), M ± SD	3.08 ± 0.50	3.06 ± 0.52	3.10 ± 0.44	3.19 ± 0.46	0.591
DES length (mm), M ± SD	23.7 ± 7.2	24.0 ± 7.3	23.2 ± 7.3	22.9 ± 6.9	0.792
DES inflation pressure (atm), M ± SD	13.0 ± 2.5	12.9 ± 2.5	13.4 ± 2.9	13.2 ± 2.0	0.649
Procedural time (min), M ± SD	100.0 ± 38.6	99.4 ± 39.5	102.4 ± 33.9	99.3 ± 41.4	0.870
Contrast medium used (mL), M ± SD	252.2 ± 76.4	254.3 ± 80.1	249.8 ± 63.4	246.2 ± 77.1	0.797

Pre‐PCI					

RLD (mm), M ± SD	2.84 ± 0.48	2.84 ± 0.52	2.89 ± 0.46	2.81 ± 0.47	0.058
MLD (mm), M ± SD	0.82 ± 0.36	0.81 ± 0.37	0.82 ± 0.35	0.87 ± 0.29	0.601
DS (%), M ± SD	69.4 ± 10.7	69.8 ± 10.9	68.5 ± 10.7	68.7 ± 9.6	0.622

Post‐PCI					

RLD (mm), M ± SD	2.89 ± 0.51	2.88 ± 0.53	2.92 ± 0.50	2.86 ± 0.49	0.065
MLD (mm), M ± SD	2.35 ± 0.35	2.37 ± 0.36	2.28 ± 0.33	2.38 ± 0.35	0.241
DS (%), M ± SD	22.9 ± 7.0	23.1 ± 6.9	22.4 ± 7.7	22.5 ± 6.2	0.722
Acute gain lesion (mm), M ± SD	1.53 ± 0.49	1.55 ± 0.48	1.47 ± 0.52	1.50 ± 0.49	0.455

Abbreviations: DCB, drug‐coated balloon; DES, drug‐eluting stent; DS, diameter stenosis; LAD, left anterior descending artery; LCX, left circumflex artery; M ± SD, mean ± standard deviation; MLD, minimal lumen diameter; NA, neoatherosclerosis; PCI, percutaneous coronary intervention; RCA, right coronary artery; RLD, reference lumen diameter.

### 3.2. OCT Findings

Details of the OCT findings are shown in Table 3. The prevalence of intrastent plaque rupture, TCFA, macrophage, and thrombus was lower in calcified NA than in lipidic and mixed NA. The reference lumen area and minimum lumen area of pre‐PCI were larger in calcified NA than in those with lipidic and mixed NA. AS of pre‐PCI was the largest among the three groups. The AS, acute lumen area gain, and minimum lumen area after PCI were similar in the three groups.

**Table 3:** Optical coherence tomography characteristics according to the different patterns of neoatherosclerosis.

	Total (*n* = 288)	Lipidic NA (*n* = 188)	Calcified NA (*n* = 42)	Mixed NA (*n* = 58)	*p* value
Intrastent plaque rupture, *n* (%)	76 (26.4)	58 (30.9)	0 (0)	18 (31.0)	< 0.001
TCFA‐like pattern, *n* (%)	106 (36.8)	77 (41.0)	0 (0)	29 (50.0)	< 0.001
Intraintima microvessels, *n* (%)	150 (52.1)	92 (48.9)	21 (50.0)	37 (63.8)	0.135
Macrophage, *n* (%)	100 (34.7)	72 (38.3)	0 (0)	28 (48.3)	< 0.001
Thrombus, *n* (%)	78 (27.1)	54 (28.7)	1 (2.4)	23 (39.7)	< 0.001

Pre‐PCI					

Reference lumen area (mm^2^), M ± SD	6.54 ± 2.21	6.48 ± 2.24	6.78 ± 2.19	6.46 ± 2.22	0.008
AS (%), M ± SD	79.1 ± 9.3	79.7 ± 9.1	75.3 ± 8.2	81.3 ± 10.2	0.002
MLA, M (IQR) (mm^2^)	1.35 ± 0.70	1.28 ± 0.66	1.65 ± 0.57	1.23 ± 0.92	0.001

Post‐PCI					

Reference lumen area (mm^2^), M ± SD	6.73 ± 2.32	6.67 ± 2.33	6.93 ± 2.26	6.76 ± 2.35	0.039
AS (%), M ± SD	34.3 ± 5.6	33.8 ± 5.4	31.6 ± 5.8	35.4 ± 5.5	0.168
MLA, M (IQR) (mm^2^)	4.52 (4.03, 6.03)	4.52 (3.94, 6.06)	4.89 (3.87, 6.35)	4.50 (4.50, 5.23)	0.669
Acute lumen area gain, M ± SD (mm^2^)	3.58 (2.63, 4.55)	3.58 (2.63, 4.69)	3.19 (2.28, 4.53)	3.58 (3.31, 4.04)	0.551

Abbreviations: AS, area stenosis; M (IQR), median (interquartile range); M ± SD, mean ± standard deviation; MLA, minimal lumen area; NA, neoatherosclerosis; PCI, percutaneous coronary intervention; TCFA, thin cap fibroatheroma.

### 3.3. Clinical Outcomes

**Figure 3:**
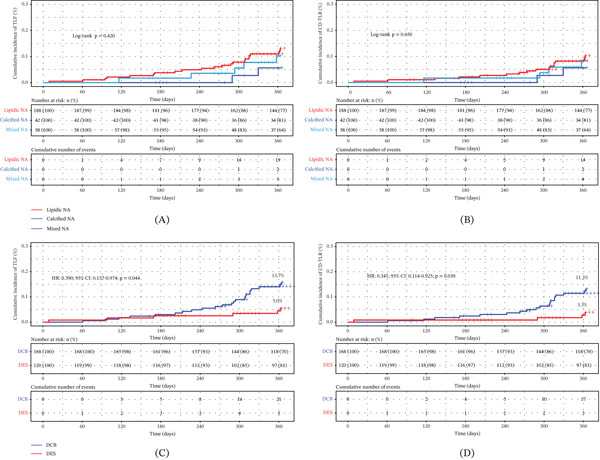
Kaplan–Meier curves for 1‐year clinical outcomes according to neoatherosclerosis (NA) patterns and treatment modality. (A) Cumulative incidence of TLF among lipidic, calcified, and mixed NA groups. (B) Cumulative incidence of CD‐TLR among the three NA patterns. (C) Comparison of TLF between DCB angioplasty and DES reimplantation. (D) Comparison of CD‐TLR between DCB and DES. CD‐TLR, clinically driven target lesion revascularization; DCB, drug‐coated balloon; DES, drug‐eluting stent; TLF, target lesion failure.

Clinical events and the 1‐year primary and secondary endpoints are summarized in Table S1. No significant difference was found in the prevalence of 1‐year TLF (*p* = 0.420) and CD‐TLR (*p* = 0.650) among the three groups (Figure 3A,B). Given the limited number of outcome events, a reduced multivariable Cox regression model was constructed to minimize the risk of overfitting, including a restricted set of clinically relevant covariates (age, hypertension, diabetes mellitus, treatment modality, and NA patterns). In this model, NA patterns were not significantly associated with TLF (adjusted *p* = 0.572 and 0.403 for comparisons) or CD‐TLR (adjusted *p* = 0.700 and 0.565), whereas treatment modality remained significantly associated with both TLF (HR: 0.390, 95% CI: 0.157–0.974, *p* = 0.044) and CD‐TLR (HR: 0.341, 95% CI: 0.114–0.925, *p* = 0.039) (Figure 3C,D and Tables S2 and S4).

To further assess the robustness of these findings, sensitivity analyses using alternative model specifications were performed, yielding consistent results. Specifically, DES implantation remained associated with a lower risk of TLF (HR: 0.399, 95% CI: 0.159–0.944, *p* = 0.041) and CD‐TLR (HR: 0.353, 95% CI: 0.117–0.916, *p* = 0.037) compared with DCB angioplasty (Tables S3 and S5).

**Figure 4:**
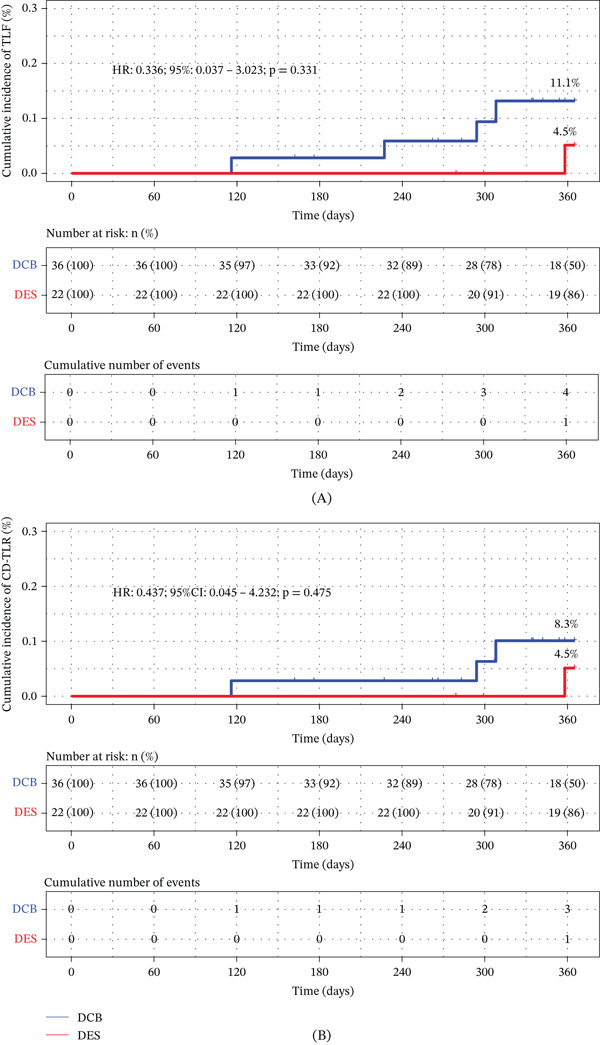
Kaplan–Meier curves for 1‐year clinical outcomes according to treatment type (DES vs. DCB) in a patient with lipidic NA. (A) Comparison of TLF between DCB angioplasty and DES reimplantation. (B) Comparison of CD‐TLR between DCB and DES. CD‐TLR, clinically driven target lesion revascularization; TLF, target lesion failure.

In subgroup analyses, among patients with lipidic NA, DES reimplantation was associated with a significantly lower risk of TLF and CD‐TLR compared with DCB angioplasty after adjustment for age, hypertension, diabetes mellitus, and treatment modality (TLF: HR: 0.287, 95% CI: 0.097–0.849, *p* = 0.024; CD‐TLR: HR: 0.171, 95% CI: 0.039–0.750, *p* = 0.019) (Figure 4 and Table 4). In contrast, no statistically significant differences between DES and DCB were observed in calcified and mixed NA subgroups (Supporting Information 1: Figure S1 and Supporting Information 2: Figure S2 and Table 4). Given the relatively small sample sizes and limited number of events in these subgroups, these negative findings should be interpreted with caution due to the potential for insufficient statistical power. Importantly, no significant interaction was observed between NA patterns and treatment modality (*p* for interaction = 0.543), suggesting that the apparent differences across NA phenotypes should be interpreted cautiously.

**Table 4:** Clinical outcomes at 1 year between the treatment modalities in patients with different patterns of neoatherosclerosis.

**Patients with lipidic NA**				

**Clinical outcomes**	**Drug-eluting stent (** **n** = 87**)**	**Drug-coated balloon (** **n** = 107**)**	**Hazard ratio (95% CI)**	**p** **value**
TLF	4 (4.9)	18 (16.8)	0.287 (0.097, 0.849)	0.024
Cardiac death	2 (2.5)	4 (3.7)	0.591 (0.099, 3.316)	0.563
Nonfatal MI	1 (1.2)	5 (4.7)	0.580 (0.054, 6.243)	0.653
CD‐TLR	2 (2.5)	15 (14.0)	0.171 (0.039, 0.750)	0.019

**Patients with calcified NA**				

**Clinical outcomes**	**Drug-eluting stent (** **n** = 25**)**	**Drug-coated balloon (** **n** = 17**)**	**Hazard ratio (95% CI)**	**p** **value**
TLF	1 (5.9)	1 (4.0)	2.016 (0.117, 34.771)	0.630
Cardiac death	—	—	—	—
Nonfatal MI	—	—	—	—
CD‐TLR	1 (5.9)	1 (4.0)	2.016 (0.117, 34.771)	0.630

**Patients with mixed NA**				

**Clinical outcomes**	**Drug-eluting stent (** **n** = 22**)**	**Drug-coated balloon (** **n** = 36**)**	**Hazard ratio (95% CI)**	**p** **value**
TLF	1 (4.5)	4 (11.1)	0.336 (0.037–3.023)	0.331
Cardiac death	0 (0)	1 (2.8)	—	—
Nonfatal MI	0 (0)	1 (2.8)	—	—
CD‐TLR	1 (4.5)	3 (8.3)	0.437 (0.045–4.232)	0.475

*Note:* Adjusted for age, hypertension, diabetes mellitus, dyslipidemia, and treatment modalities.

Abbreviations: CD‐TLR, clinically driven target lesion revascularization; MI, myocardial infarction; NA, neoatherosclerosis; TLF, target lesion failure.

After PSM, 158 patients (79 pairs) were included in the matched cohort. Baseline clinical, angiographic, and OCT‐derived characteristics were well balanced between the DES and DCB groups (Tables S6, S7, and S8). In the propensity score–matched cohort, clinical outcomes were similar according to NA patterns (TLF *p* = 0.820 and CD‐TLR *p* = 0.880) (Supporting Information 3: Figure S3), while treatment modality remained significantly associated with both TLF and CD‐TLR (Supporting Information 4: Figure S4). Among patients with lipidic NA, DES treatment was associated with a significantly lower risk of TLF compared with DCB angioplasty (HR: 0.121, 95% CI: 0.015–0.978; *p* = 0.048). Similarly, the risk of CD‐TLR was seemingly reduced in the DES group (HR: 0.140, 95% CI: 0.017–1.152; *p* = 0.067) (Supporting Information 5: Figure S5 and Table S9). In contrast, no significant differences in TLF or CD‐TLR were observed between DES and DCB in patients with calcified or mixed NA (Supporting Information 6: Figure S6 and Supporting Information S2: Figure S7).

## 4. Discussion

The main findings of the current study can be summarized as follows: (1) no significant differences were found in TLF or CD‐TLR among the lipidic, calcified, and mixed NA groups in ISR patients undergoing treatment with DCB or DES; (2) repeat DES implantation was associated with a lower risk of TLF and CD‐TLR compared with DCB angioplasty. In subgroup analyses, a similar association was observed in patients with lipidic NA; however, no statistically significant interaction between treatment modality and NA pattern was identified. Therefore, these subgroup findings should be interpreted with caution and should not be considered as definitive evidence of differential treatment effects across NA subtypes.

Importantly, the present study focused on clinical outcomes in patients with pre‐existing OCT‐defined NA undergoing repeat PCI rather than the development of NA itself. Therefore, the 1‐year follow‐up period was selected to capture early treatment failure after intervention. Previous evidence suggests that most CD‐TLR events after PCI for ISR occur within the first year, supporting the clinical relevance of this time frame [20]. However, NA is a progressive pathological process, and late adverse events—such as recurrent NA or delayed restenosis, particularly after repeat DES implantation—may not be fully captured within 1 year. Thus, the present findings should be interpreted as reflecting early‐ to mid‐term outcomes, and longer term follow‐up is warranted to evaluate the durability of DES versus DCB strategies.

NA is a major cause of stent failure. Previous investigations indicated that patients with NA had a worse clinical prognosis than those without [8, 9]. Based on different patterns of NA, Chen et al. [12] found that only lipidic NA, not the calcified form, was associated with poor clinical outcomes following repeat revascularization. In the current study, NA was divided into lipidic, calcified, and mixed patterns. No obvious differences were found in the incidence of TLF and CD‐TLR among the three types. Actually, our findings indicate that calcified NA may be associated with more favorable clinical outcomes, which may be attributed to the limited sample size.

Current guidelines strongly recommend either DES reimplantation or DCB angioplasty as the primary treatment of ISR [2]. However, there is currently no consensus on the optimal scenarios for choosing stent reimplantation versus DCB intervention. Researches focused on the correlation between OCT‐defined neointimal patterns and clinical outcomes were relatively scant [21, 22]. Xhepa et al. [21] reported that DES reimplantation rather than DCB angioplasty was more suitable for ISR patients with high inhomogeneity. In the current study, we investigated the clinical prognosis between treatment modalities and different patterns of NA. The results indicated DES reimplantation reduced the incidence of TLF compared with DCB angioplasty in lipidic NA lesions. The trend existed in mixed pattern lesions, and it seemed that as the sample size increased, this difference would become more pronounced. NA is defined by the presence of lipid‐accumulating foamy macrophages in the neointima, which may be accompanied by necrotic core development and/or calcification [23]. In the progression of atherosclerosis, the balance between proinflammatory and inflammation‐resolving mechanisms in lipid‐driven inflammation plays a critical role [24], while calcification may be attributed to apoptosis of foamy macrophages or smooth muscle cells or the calcification of the collagen, extracellular matrix, and smooth muscle cells [25]. Therefore, it is conceivable that the inflammation in lipidic NA lesions is more severe than that in calcified lesions. The effectiveness of DCB therapy depends on the efficient initial delivery and prolonged tissue retention of the antiproliferative drug, which may be more suitable for those lesions with relatively active proliferation of smooth muscle cells. In this regard, repeat DES implantation may suppress both smooth muscle cell proliferation and inflammatory activation, which may account for the result of this study.

Moreover, lipidic NA frequently exhibits features analogous to TCFA, including a thin fibrous cap, large lipid pool, and prominent macrophage infiltration. These characteristics have been well established as hallmarks of plaque vulnerability in native coronary artery disease. In the setting of ISR, TCFA‐like NA may contribute to adverse outcomes through several mechanisms. First, these lesions are prone to plaque destabilization, including rupture or erosion, which may trigger thrombus formation and acute luminal compromise. Pathological and imaging studies have demonstrated that NA is an important substrate for late stent failure, including restenosis and very late stent thrombosis [25]. Second, even in the absence of overt plaque rupture, lipid‐rich neointima is characterized by persistent inflammation and increased biological activity, which may accelerate neointimal proliferation and progressive luminal narrowing. Previous studies have shown that lipidic neointima and TCFA‐like patterns are associated with higher rates of restenosis and repeat revascularization [26, 27]. Therefore, TCFA‐like morphology in NA provides a plausible mechanistic link between imaging‐defined vulnerability and CD‐TLR through both acute destabilization and chronic progression pathways.

However, beyond these biological considerations, the interaction between stent struts and the underlying neointimal tissue may play a critical role in determining treatment efficacy. On one hand, calcified NA, due to its rigid and noncompliant nature, may limit stent expansion and reduce strut embedding into the vessel wall. In contrast, lipid‐rich neointima is more deformable, allowing deeper strut penetration and closer contact between the stent surface and surrounding tissue. Previous studies have demonstrated that neointimal coverage overlying stent struts is significantly thinner in calcified lesions compared with lipid‐rich lesions, suggesting impaired healing and reduced biological response in calcified tissue [28]. On the other hand, lipid‐rich NA is characterized by the accumulation of foamy macrophages and necrotic core components, with early lesions typically forming in the peristrut region, reflecting a localized inflammatory response driven by stent–tissue interaction [29]. This peristrut lipid‐rich microenvironment is characterized by increased macrophage infiltration and neovascularization, reflecting an enhanced inflammatory state that may increase biological responsiveness to antiproliferative therapy [29]. In contrast, calcified neointima consists of a dense, mineralized extracellular matrix with low permeability, which may act as a physical barrier to drug penetration and reduce effective drug delivery into the vessel wall. Taken together, these findings suggest that the superior efficacy of DES in lipidic NA lesions is likely driven by a combination of enhanced biological susceptibility (inflammation and smooth muscle cell proliferation) and more favorable mechanical and pharmacokinetic conditions for drug delivery. In contrast, calcified neointima, characterized by lower inflammatory activity, limited strut embedding, and reduced drug permeability, may attenuate the relative benefit of DES, resulting in comparable outcomes between DES and DCB therapies.

The greatest advantage of DCB is its excellent angiographic results without the need for additional stent layers. Therefore, it is feasible to utilize DCB for the treatment of patients with ISR whenever possible. Due to their favorable pharmacokinetic properties, paclitaxel‐coated balloons (PCBs) provide the majority of the evidence supporting DCBs for the treatment of ISR in contemporary practice [30, 31]. Sirolimus may offer advantages over paclitaxel in terms of its antirestenotic and anti‐inflammatory efficacy. However, due to the low lipophilicity property of sirolimus, current studies comparing sirolimus‐coated balloons (SCBs) and PCBs for the treatment of ISR lesions have not demonstrated significant superiority [32, 33]. Moreover, the ISAR‐DESIRE 4 [34] study demonstrated that neointimal modification with scoring balloon plus DCB provided better antirestenotic efficacy than DCB standard therapy. And a possible reason is that moderate localized injury may enhance the penetration and subsequent retention of the antirestenotic agent. Therefore, for lipid‐rich NA lesions, which present a highly inflammatory state, the clinical outcomes may be likely to improve further with the continuous iteration of new‐generation DCBs combined with appropriate neointimal modification.

## 5. Study Limitations

There were several limitations in this study. First, this was a retrospective, single‐center study, and the findings should be considered exploratory. Second, the treatment strategy was determined at the operator′s discretion, introducing potential selection bias. To mitigate this, PSM was performed to balance baseline clinical, angiographic, and OCT characteristics between treatment groups. The main findings remained consistent after matching and were further supported by multivariable Cox regression with cluster adjustment and subgroup analyses, suggesting the robustness of the results. Nevertheless, residual confounding due to unmeasured variables cannot be completely excluded. Third, the overall sample size was limited, with imbalance across NA subgroups, which may have reduced statistical power to detect differences between groups. Finally, as discussed above, the relatively short follow‐up duration limits assessment of long‐term outcomes, and future studies with extended follow‐up are warranted.

## 6. Conclusions

In this study, no significant differences in clinical outcomes were observed across different NA patterns. DES reimplantation was associated with a lower risk of TLF and CD‐TLR compared with DCB angioplasty. Although numerically favorable outcomes were observed with DES in patients with lipidic NA, no significant interaction between treatment modality and NA pattern was identified. Therefore, these subgroup findings should be interpreted with caution.

## Author Contributions

Bei Shi, Yongchao Zhao, and Youcheng Shen contributed to the study conception and design. Material preparation was performed by Youcheng Shen, Ning Gu, and Zhijiang Liu. Data on patients′ demographic collection was performed by Qianhang Xia and Youcheng Shen. The analysis of the coronary angiography was performed by Wei Zhang and Xi Wang. The analysis of OCT images was performed by Chancui Deng and Zhijiang Liu. The first draft of the manuscript was written by Bei Shi, Yongchao Zhao, and Youcheng Shen. Youcheng Shen, Ning Gu, and Zhijiang Liu contributed to the work equally and should be regarded as cofirst authors.

## Funding

This work was supported by grants from the Guizhou Provincial Department of Education Young Scientific and Technological Elite Talent Program (QianJiaoJi [2024] 332) and the Guizhou Provincial Basic Research Program (Natural Science) (Qiankehe Foundation‐ZK [2024] Key 068 and Qiankehe Foundation‐ZK [2022] General 671).

## Disclosure

All authors commented on previous versions of the manuscript. All authors read and approved the final manuscript.

## Ethics Statement

This study was conducted in accordance with the principles of the Declaration of Helsinki. Approval was granted by the Ethics Committee of Zunyi Medical University. As this study had a retrospective design and all procedures were performed based on clinical necessity, with consent obtained prior to each procedure, the requirement for informed consent was waived.

## Conflicts of Interest

The authors declare no conflicts of interest.

## Supporting Information

Additional supporting information can be found online in the Supporting Information section.

## Supporting information


**Supporting Information 1** Figure S1: Kaplan–Meier curves for 1‐year clinical outcomes according to treatment type (DES vs. DCB) in a patient with calcified NA. (A) Comparison of TLF between DCB angioplasty and DES reimplantation. (B) Comparison of CD‐TLR between DCB and DES. CD‐TLR, clinically driven target lesion revascularization; TLF, target lesion failure.

**Supporting Information 2** Figure S2: Kaplan–Meier curves for 1‐year clinical outcomes according to treatment type (DES vs. DCB) in a patient with mixed NA. (A) Comparison of TLF between DCB angioplasty and DES reimplantation. (B) Comparison of CD‐TLR between DCB and DES. CD‐TLR, clinically driven target lesion revascularization; TLF, target lesion failure.

**Supporting Information 3** Figure S3: Kaplan–Meier curves for 1‐year clinical outcomes among the different patterns of neoatherosclerosis after propensity score matching. (A) Cumulative incidence of TLF among lipidic, calcified, and mixed NA groups. (B) Cumulative incidence of CD‐TLR among the three NA patterns. CD‐TLR, clinically driven target lesion revascularization; TLF, target lesion failure.

**Supporting Information 4** Figure S4: Kaplan–Meier curves for 1‐year clinical outcomes according to treatment type (DES vs. DCB) after propensity score matching. (A) Comparison of TLF between DCB angioplasty and DES reimplantation. (B) Comparison of CD‐TLR between DCB and DES. CD‐TLR, clinically driven target lesion revascularization; TLF, target lesion failure.

**Supporting Information 5** Figure S5: Kaplan–Meier curves for 1‐year clinical outcomes according to treatment type (DES vs. DCB) in a patient with lipidic NA after propensity score matching. (A) Comparison of TLF between DCB angioplasty and DES reimplantation. (B) Comparison of CD‐TLR between DCB and DES. CD‐TLR, clinically driven target lesion revascularization; TLF, target lesion failure.

**Supporting Information 6** Figure S6: Kaplan–Meier curves for 1‐year clinical outcomes according to treatment type (DES vs. DCB) in a patient with calcified NA after propensity score matching. (A) Comparison of TLF between DCB angioplasty and DES reimplantation. (B) Comparison of CD‐TLR between DCB and DES. CD‐TLR, clinically driven target lesion revascularization; TLF, target lesion failure.

**Supporting Information 7** Figure S7: Kaplan–Meier curves for 1‐year clinical outcomes according to treatment type (DES vs. DCB) in a patient with mixed NA after propensity score matching. (A) Comparison of TLF between DCB angioplasty and DES reimplantation. (B) Comparison of CD‐TLR between DCB and DES. CD‐TLR, clinically driven target lesion revascularization; TLF, target lesion failure.

## Data Availability

All data generated and analyzed during this study are included in this published article.
